# How do family members perceive re-integration of male state patients into their families in South Africa? A qualitative analysis

**DOI:** 10.4102/sajpsychiatry.v26i0.1450

**Published:** 2020-10-21

**Authors:** Ndivhaleni R. Lavhelani, Mary Maluleke, Mulatedzi P. Mulaudzi, Thingahangwi C. Masutha, Takalani E. Makhubele, Duppy Manyuma, Muofheni Nemathaga, Ndiambani A. Makhavhu, Martha L. Kharivhe, Takalani E. Mbedzi, Muvhango R. Ramovha, Dorah U. Ramathuba, Mutshinyalo L. Netshikweta

**Affiliations:** 1 Limpopo Department of Health, Hayani Psychiatric Hospital, Vhembe District, Thohoyandou, South Africa; 2 Department of Advanced Nursing Science, School of Health, University of Venda, Thohoyandou, South Africa; 3 Department of Health, Faculty of Nursing, Limpopo College of Nursing, Thohoyandou, South Africa; 4 Limpopo Department of Health, Tshilwavhusiku Clinic, Vhembe District, Thohoyandou, South Africa; 5 United Nations High Commission for Refugees (UNHCR) Programme, Pretoria, South Africa; 6 Limpopo Department of Health, Tshilidzini Hospital, Thohoyandou, South Africa; 7 Limpopo Department of Health, Louis Trichardt Memorial Hospital, Thohoyandou, South Africa; 8 Limpopo Department of Health, Vhembe District, Thohoyandou, South Africa

**Keywords:** family, health establishment, re-integration, state patient, support

## Abstract

**Background:**

State patients are admitted to a psychiatric hospital after being declared as such by the magistrate courts as a result of not found fit to stand trial for the offence they had committed. After successful rehabilitation of state patients at the psychiatric hospital, they need to be re-integrated into their families. Family members’ perceptions regarding re-integration of male state patients are not largely explored in the scientific body of knowledge.

**Aim:**

The aim of the study was to determine the perceptions of family members regarding reintegration of male state patients into their families.

**Setting:**

This study was conducted in Vhembe District of Limpopo province, South Africa.

**Method:**

A qualitative approach using explorative, descriptive and contextual designs was adopted. In-depth interviews were conducted with 10 family members who were purposefully sampled, and data were thematically analysed using Tech Open Coding method.

**Results:**

Three themes emerged, namely, family members understand re-integration; family members’ expectations from a mental healthcare user; and threat to re-integration as perceived by family members.

**Conclusion:**

Data revealed that family members have varied perceptions regarding re-integration. These perceptions were based on the behaviour displayed or an offence committed by the mental healthcare user before admission and how participants experienced it. The study recommends that an investigation is to be conducted on the kind of support family members need regarding re-integration of state patients into their families.

## Background

State patients are admitted to a psychiatric hospital after being declared as such by magistrate courts after being found not fit to stand trial for the offence they had committed. Over the past few years, mental healthcare has become increasingly rendered in hospitals globally, with recovery as a key to the treatment of mentally ill patients. Therefore, supporting family members of state patients in mental healthcare, in order to build trust, are supposed to interact with mental healthcare practitioners and discuss how mental illness affects all areas of a person’s life. As a result of de-institutionalisation and lack of adequate community support for state patients, family members often provide support to patients with psychiatric disorders, such as schizophrenia, bipolar and other major depressive disorders.^1^ In the United States of America (USA), estimates have shown that care is provided by family members to one-third to two-thirds of patients with long-term psychiatric disabilities. Research has shown that 40% of patients with schizophrenia live with their relatives; in China, also more than 90% of people suffering with schizophrenia live with their families.^2^

In South Africa, the National Mental Health Policy Framework and Strategic Plan fills a critical gap in South African national policy framework and gives added substance to the *Mental Health Care Act*, No. 17 of 2002. This act sets up legal framework for a primary community- and healthcare-based mental health system on the fundaments of human rights. The notion that people with mental illness or intellectual disability are banished from communities and institutions is not only outdated but also inhumane. Modern mental health policy has carried out the re-integration of mental healthcare into general health services and the development of community-based care at the centre of interventions, whilst recognising that a small minority still require specialised in-patient care for varying lengths of time as per the National Mental Health Policy Framework and Strategic Plan document 2013.^3^

The findings of a study conducted by Giacco et al.^4^ emphasise that patients and families have a desire for information regarding mental illness so that they should take informed decisions about the care of mentally ill patients. Furthermore, the desire for information by mentally ill patients is associated with variables, which may change over time, such as symptoms severity and therapeutic relationship. This finding supported the researchers’ aim of the current study to explore the perceptions of family members regarding re-integration of male state patients.

Another study conducted by Berthelsen et al.^5^ has identified that in mental health, support to the family members and relatives of state patients is very crucial.

However, in order to fast-track treatment models, this study has reflected that knowledge is required to strengthen their involvement. During the study, Hayani Psychiatric Hospital was the only designated psychiatric hospital, with a functional maximum-security ward infrastructure that renders mental health services to male state patients in the Vhembe district of Limpopo province, in South Africa. After successful rehabilitation of state patients at Hayani Psychiatric Hospital, they are to be re-integrated into their families. The main author (N.R.L.) has since observed that most stable male state patients are rejected by their families when they are due for leave of absence or discharged to go home. This is supported by the study conducted by Berthelsen et al.,^5^ who found that mental healthcare users (MHCUs) experience rejection by their family members who often display negative attitudes towards acceptance of MHCUs at home. Family members’ perceptions regarding reintegration of male state patients have not been largely explored in the body of knowledge. As a step to address this evidence–practice gap, this study aimed to determine and document perceptions of family members regarding re-integration of male state patients into their families.

This study explored family members’ perceptions regarding re-integration of male state patients into their families.

## Methods

### Study design

To achieve the aim of this study, a qualitative approach using explorative, descriptive and contextual design was adopted as described in the literature.^6,7,8,9^

### Study settings

This study was conducted in the Limpopo province of South Africa. Limpopo province is divided into five districts: Vhembe, Mopani, Capricorn, Sekhukhune and Waterberg. Limpopo province mostly has rural settings, except for some parts of Capricorn district. This study was conducted in Vhembe district, where Hayani Psychiatric Hospital is located. The study involved the family members of male state patients at their homes where they stay with these patients, although the patients were still admitted to Hayani Psychiatric Hospital when the study was conducted. Other districts were not chosen because they do not have a specialised psychiatric hospital similar to Hayani with a functional maximum-security ward infrastructure, where many state patients who need to be re-integrated with their families could be admitted.

### Recruitment and sampling

A non-probability purposive sampling was preferred as it is ‘based on the judgment of the researcher regarding participants knowledgeable about the question at hand’.^9,10^ Sampling occurred in three stages: sampling of district, hospital and participants.

#### District

The Vhembe district of Limpopo province in South Africa was purposively selected for this study, as it was the only district in Limpopo province with a specialised licensed mental hospital, with a functional maximum-security ward infrastructure rendering forensic mental health services (admitting males who committed crimes). No other district in Limpopo province has such a specialised licensed mental hospital.

#### Hospital

Within Vhembe district, this specialised licensed mental hospital was purposively sampled because it was the only hospital with a functional maximum-security ward where male state patients are admitted. The hospital had 20 male state patients rejected by their relatives at the time of data collection. All hospitals without functional maximum-security wards infrastructure in or outside Vhembe district were not part of the study.

#### Participants

All Venda-speaking family members of male state patients who were admitted to Hayani Psychiatric Hospital were purposively sampled. A permission letter was granted by the hospital’s chief executive officer to access the family members’ names that were found in the hospital’s admission register. Only those with complete contact details were allocated listed numbers. Those without complete contact details were not listed, and only listed persons were selected purposively. The total number of listed persons was 37 and they were recruited telephonically. Out of the 37 recruited, 16 family members agreed to participate in the study and only 10 participated in the study owing to data saturation. Family members with the following details were excluded from the study: not related biologically to the male state patient, had incomplete contact details on the hospital register, not staying with the male state patient, residing outside Vhembe district, not Tshivenda-speaking and not willing to participate in the study.

### Data collection

Data collection involved preparation of participants and data collection instrument.

#### Preparation of participants

Family members of male state patients were recruited telephonically to participate in the study after the study was explained to them. The reason for recruiting them for the study was explained to family members because they were experts in the topic of the study, and that it would provide them a platform to talk about their views regarding re-integrating of male state patients with their relatives. This was followed by making telephonic appointments at their homes at the time convenient to them. Tentative dates were given and the time and venue were agreed upon by the participants. Contact numbers of the researcher and those of participants were exchanged to facilitate any changes in the agreed arrangements.

#### Data collection instruments

Data were collected through in-depth individual interviews with family members. All interviews were directed by the following central question, which was followed by further probing questions during the interviews: *as a relative of a patient who committed a crime, could you please share with me your perceptions regarding re-integrating him into your family?*

The interviews were audio-recorded, then transcribed verbatim and finally translated into English for analysis.

### Data analysis

The transcripts were analysed by the authors collectively using qualitative content of Tech Open Coding Method^11^ as follows: Transcribed data were read for several times to gain overall meaning of the responses given by family members. The data were then arranged into categories and subcategories and labelled using the actual words and language of family members. Three themes emerged after data were arranged, which were the major findings of the study. Furthermore, authors cross-checked and interpreted the identified themes, wherein they explained what was learnt about family members’ perceptions regarding reintegration of male state patients into their families.

### Ethical consideration

Ethical clearance to conduct the study was obtained from the University of Venda Research Ethics Committee (ethical clearance number: SHS/18/PDC/06/0905, May 2018), Limpopo Provincial Department of Health and Vhembe district Department of Health, and the selected hospital issued a letter to allow the researchers to have family members’ contact details in order to recruit participants from their homes. All participants were provided with information sheets and consent forms before they were recruited into the study. Participants were informed that they were under no obligation to participate in the study, but if they do so, they had the right to withdraw at any stage of the study. Complete operation of conducting interviews was explained to participants, including functioning of audio tape, so that participants were free to stop it for some reason.

## Results

### Participants’ characteristics

The sample comprised 10 participants recruited from 10 family units; this sample size depended on the saturation of data. Family units were numbered from 1 to 10, and they all had the responsibility for taking care of male state patients when released from the hospital. Therefore, in order to have demographic information, the gender of participant and relation with state patient were noted on first contact. Below is the discussion and illustration of each interview.

### Gender information of participants

**FIGURE 1:**
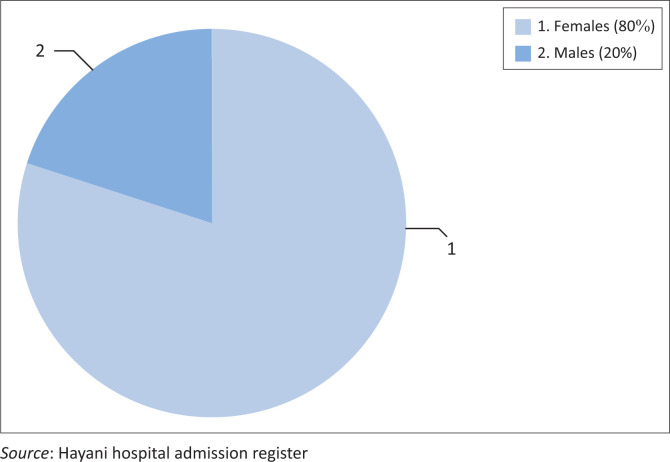
Gender information of the study participants.

Data revealed that the majority of participants (8; 80%) were women, whilst two (20%) participants were men as illustrated in Table 1 and Figure 1. This is common in Limpopo rural areas because the majority of men had migrated to other provinces in search of jobs, and households were run by women. This is supported by Limpopo province Department of Health’s 5-year ( 2015–2020) Strategic Plan report (2015), which stated that Limpopo province’s unemployment rate was estimated at 18.1%. Furthermore, women constituted the majority, making up to 52.3% (2.73 million) of province’s population.

**TABLE 1:** Gender information of the study participants.

Gender	Frequency	Percentage
Females	8	80
Males	2	20
**Total**	**10**	**100**

*Source*: Hayani hospital admission register

### Relationship information of participants and the mental healthcare user

**FIGURE 2:**
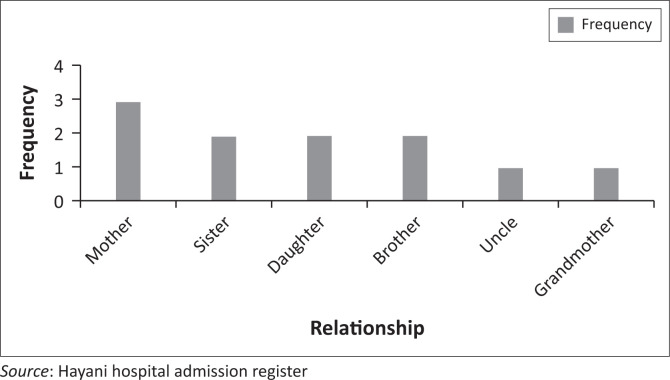
Frequency relationship information of participants and the mental healthcare user.

**FIGURE 3:**
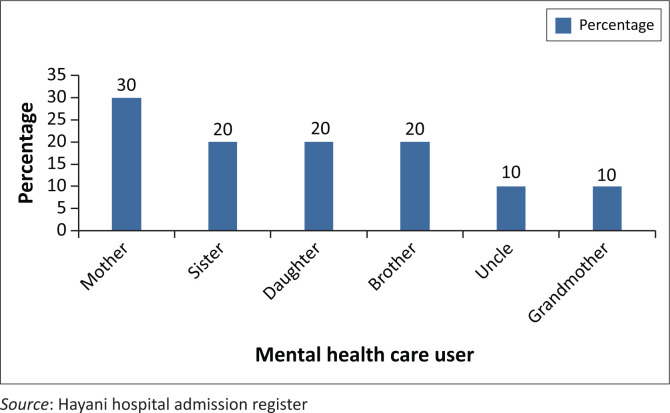
Percentage relationship information of participants and the mental healthcare user in percentage.

Regarding the relationship of participants with MHCU, the following details emerged: out of 10 participants, three (30%) were mothers, two (20%) were sisters, two (20%) were daughters, and one each was brother (10%), uncle (10%) and grandmother (10%) as shown in Table 2 and Figures 2 and 3. All were biological relatives of male state patients. This is in line with the Social Ecological Model of Human Behaviour, which states human behaviour to be multi-dimensional in individuals and relations.

**TABLE 2:** Relationship information of participants and the mental healthcare user.

Relationship	Frequency	Percentage
Mother	3	30
Sister	2	20
Daughter	2	20
Brother	2	20
Uncle	1	10
Grandmother	1	10
**Total**	**10**	**100**

*Source*: Hayani hospital admission register

## Presentation of findings

Data analysis revealed three themes regarding how family members experienced re-integration of a state patient into their families. The participant direct quotes are verified by three identifiers, namely, Participant number (P), gender and relationship.

### Theme 1: Family members understand re-integration

Most participants indicated that they understand and accept re-integration; therefore, they would like their male state patient to return home. The following statements attest to participants’ perceptive:

‘Myself as a sister to him, I see [*him*] coming back home as a good thing. Myself as his sister, I feel that as a human being, he might now be tired of staying in the hospital for a long time as a mentally ill patient who had stayed in the hospital for a long time …’. (P8, female, sister)

‘Myself I like it as if I reject him it will be like I am saying that the hospital should take responsibility for caring of my son. I have been given my son by God and He want to see if we accept him back home. If I reject him, it will be like I am rejecting my God …’. (P7, female, grandmother)

‘My perception regarding this question is that I see it as important for those patients to be released back into their families. Myself, I like it. My father usually comes home for short periods. He then returns to the hospital when we realised that things are not right with …’. (P10, female, daughter)

Some family members confessed their love for their male state patients as they narrated about the kind of love towards the patient in terms of either being a mother, daughter or brother. The following statements attest to it:

‘I love him so much, he is a gift from God. What he did was influenced by mental illness, I love my son very much, he should come back …’. (P2, female, mother)

‘I love and accept my father very much. I accept him as my father and the other family members also accept him. He is mentally ill and we will support him to take his medication until he is stable …’. (P9, female, daughter)

‘I love him as my biological brother, I will never abandon. I love him and I accept him to come back home, I understand that he is mentally ill …’. (P4, male, brother)

### Theme 2: Family members’ expectations from a mental healthcare user

Most participants indicated their expectations from MHCUs regarding re-integration. Participants stated that they would like to see their MHCU completely cured before re-integration could take place. The following statements depict family members’ expectations from MHCUs regarding re-integration.

‘If the patient is right, I agree that he must come back. My opinion of sending him to Hayani Hospital is [*was*] that it was difficult for me to stay with him. Things were not good. There was no one who can handle him except myself and my late husband who unfortunately passed away at the time of sending him to the hospital. I saw that I cannot handle him, I then requested that he be admitted. I had many trips to the SAPS and magistrate court to arrange for their assistance to me. I even came [went] to Hayani Hospital to see the doctors. He must be healed completely in order for him to come back. I mean that it should be in way that we could interact well with each other with good understanding for each other. He should be completely normal, I do not want any troubles …’. (P7, female, grandmother)

‘What I see is that if these patients are healed, they can be released to go home. I mean, he should be able to leave [*sic*] in peace with other people in peace’. (P5, male, uncle)

Some participants regarded the importance of not sending MHCUs to their homes completely; instead, they stated that MHCUs should be sent for shorter periods only:

‘I am saying that they can come home but not forever. They can be given short stays while being checked, maybe for a week, month or so. This will allow us to check whether they are completely healed or not’. (P8, female, sister)

‘They should not be discharged fully, it will [*be*] fine if they are just given some days, since their behaviour is unpredictable and are very dangerous. As much as we love and understand that they are mentally ill, we are also scared of their behaviour. Coming for few days will be proper, hey we are scared of them’. (P3, female, mother)

Some participants stated that MHCUs must comply with medication to prevent relapse. This would be achieved if healthcare professionals could check MHCUs at their homes regularly. This was depicted by the following statements:

‘As long as he takes his medication, I will accept him. The government should come and check whether the patients are continuing to take treatment and that these patients are not troubling the family members, relatives and the community at large. I also see it as important to do something that will make them busy …’. (P9, female, sister)

‘I like him to come home as my child. The important thing is that he must take his medications as instructed … The hospital should ensure that the patient get[*s*] his medication …’. (P3, female, mother)

### Theme 3: Threat to re-integration as perceived by family members

Some of the participants stated that they were scared of MHCUs. They expressed that they were victims of male state patients’ conduct, which made them fearful:

‘… Just know that I love my son very much to come back, but I am afraid of his bad conduct that if he comes back, he will cause troubles. He also hit me with his fist and two of my teeth fell (she showed where the teeth were attached in her mouth) …’. (P2, female, mother)

‘… My mother is the one who seems to be afraid for him because he locked her in the house wanting to burn the house…’. (P8, female, sister)

‘… The one who does not like him is his uncle. He has a fear for him, he killed his uncles’ father who is [*was*] his grandfather, he does not even want to hear his name mentioned; in fact, he does not want anything to do with him …’. (P1, female, sister)

One participant stated that MHCU returning home would cause instability in their family:

‘… I do not support him coming back home, it is a bad idea as he does not love his siblings, nephews and nieces. He does not love them, and I do not know where to take them as he does not love them …’. (P6, female, mother)

Some participants narrated how MHCUs’ family members are afraid of re-integration because it posed threat to them:

‘I feel that as a human being, he might now be tired of staying in the hospital for a long time as a mentally ill patient who had stayed in the hospital for a long time. I don’t know whether what the victim family had forgiven him or what are they thinking about him, if they see him coming here at home. I really don’t know?’. (P4, male, brother)

‘He was taken to prison after burning my neighbour’s house and destroying their properties calling them witches. We are no longer in good terms with my long-standing neighbours. They are prepared to do anything to him before he attacks them. I am afraid something bad will happen to him should he come back home …’. (P7, female, grandmother)

Some participants indicated that community people may take revenge of MHCU who had committed crime before he was admitted. As a result, family members assumed that re-integration would not be a success:

‘I like him to come back home but, the community say if he comes home, they will chop him as he had done a lot of damages in this village. People use not to pass freely next to our home. He usually assembles stones and throw them to educators and learners as they pass here to school’. (P5, male, uncle)

‘What my brother did is very painful to the entire village, everybody hates him, including community leaders, it is obvious that when he come[*s*] back home, they will hurt him. I am afraid’. (P8, female, sister)

## Discussion

Family members of male state patients from Vhembe district in South Africa perceived re-integration as positive because they wanted their patients to come back from hospital after rehabilitation. The findings of the study also revealed that although MHCUs committed crimes, family members had realised and accepted their re-integration into their families.

Similarly, another study had reported that generally family members were supportive; they often expressed distress at having lost their sick relative, although he or she was just not the same person as before.^12^

This is supported by yet another study which revealed that family members accepted the re-integration of their relatives having mental illness^1^ because as a result of de-institutionalisation and lack of adequate community support, family members provided support to relatives with mental illness. This support could be in the form of social support, compliance with mental and somatic health treatment, housing and numerous activities of daily living. On the other hand, a study revealed that as family members explained their process of acceptance, they expressed how they had struggled to accept condition of their mentally ill relations.^13,14,15^ They indicated that the more the victims struggled, the more their family members had problems because the victims did not receive enough support and ended up being admitted for several times because of relapse.

The present study further revealed the fear of re-integration experienced by family members. This was in line with the outcomes of other studies^16,17^ which revealed that family members experienced helplessness, fear and vulnerability caused by the aggression and violence that the patient displayed prior to admission to a psychiatric hospital. Moreover, family members felt a deep sense of fear which was an outcome of unpredictable behaviours of state patients, such as aggression, hostility, abusive language and mood fluctuations.^18,19^

This strengthens the findings alluding that psychotic episodes could occur again in the community as patients encounter specific stressors (e.g. death of a parent) or default medication.

Similar results were found about families of mentally ill patients who experienced emotional pain, guilt and concern.^20^ This was further supported by a study conducted by Monyaluoe et al.^21^ in Limpopo province, which revealed that family members of state patients experienced tension, stress, anxiety, resentment and depression with hopelessness, helplessness and destruction in their everyday lives.

Another study revealed that family members of mentally ill patients expressed their impatience towards the behaviour of their mentally ill relations.^15^ Similar results were found by a study which revealed that families of mentally ill patients experienced emotional pain, guilt and concern.^20^

This study revealed that family members had expectations from MHCU before they could accept re-integration, including healthcare professional’s home visits.^22^ It was found that family members went through a process of acceptance and received medical knowledge and assistance from health professionals. In this process families discovered strength to limit relapses of their mentally ill patients.

According to the study conducted by Lawska et al.,^22^ mentally ill patients expect to be noticed, accepted and sympathised with; hence, a supportive and accepting environment is indispensable for the optimisation of socio-professional therapy and rehabilitation of such patients. This is supported by other studies,^14,15,23^ reporting that home care could fortify and enhance the ability of natural settings to contain, support and stabilise symptomatic individuals who would otherwise require a supervised level of treatment.

Most of the participants in this study indicated threat of re-integration, which resonated from victim’s relatives and family members who were afraid of MHCU as well as the community that would revenge on MHCU. One study revealed similar results that living with and caring for an individual with psychiatric disorder seems inherently stressful because of the fear that family members experienced from mentally ill patients.^24^ Relatives of psychiatric patients reported a wide range of reactions to their situation. Overall, family members endorsed significantly higher levels of psychological distress than experienced by general population. Similarly, Monyaluoe et al.^21^ alluded that families of state patients experienced much of social rejection, which could interfere the restoration and maintenance phase of such patients. The current study had found the perceptions of family members regarding re-integration of male state patients into their families. However, the perceptions of such patients were not determined. Therefore, an investigation is recommended to determine male state patients’ views about reuniting with their families.

## Conclusion

The aim of this study was to determine the perceptions of family members regarding re-integration of state patients into their families. Data revealed that family members had varied views regarding re-integration. Some understood and accepted re-integration as an indication of love. However, the majority of families had conditions in place to welcome back MHCUs in their homes; these included that the MHCU must be mentally stable, adhere to treatment or may be on leave of absence. On the other hand, some perceived re-integration as a threat to the family, victims and the community. These perceptions were based on the behaviours displayed or the offence committed by MHCUs before being admitted for rehabilitation. As a result, MHCUs suffer from stigma of their families as well as the community. Therefore, an investigation is to be conducted on the kind of support needed by family members regarding re-integration of state patients into their families.
